# dictyExpress: a web-based platform for sequence data management and analytics in *Dictyostelium* and beyond

**DOI:** 10.1186/s12859-017-1706-9

**Published:** 2017-06-02

**Authors:** Miha Stajdohar, Rafael D. Rosengarten, Janez Kokosar, Luka Jeran, Domen Blenkus, Gad Shaulsky, Blaz Zupan

**Affiliations:** 1 Genialis d.o.o., Trzaska cesta 315, Ljubljana, 1000 Slovenia; 2 Genialis Inc., 2726 Bissonnett Street, Suite 240-374, Houston, 77005 TX USA; 3 Department of Molecular and Human Genetics, Baylor College of Medicine, 1 Baylor Plaza, Houston, 77030 TX USA; 4 Faculty of Computer and Information Science, University of Ljubljana, Večna pot 113, Ljubljana, 1000 Slovenia

**Keywords:** Bioinformatics, Visual analytics, Platform, RNA-seq, ChIP-seq, Differential gene expression

## Abstract

**Background:**

*Dictyostelium discoideum*, a soil-dwelling social amoeba, is a model for the study of numerous biological processes. Research in the field has benefited mightily from the adoption of next-generation sequencing for genomics and transcriptomics. *Dictyostelium* biologists now face the widespread challenges of analyzing and exploring high dimensional data sets to generate hypotheses and discovering novel insights.

**Results:**

We present dictyExpress (2.0), a web application designed for exploratory analysis of gene expression data, as well as data from related experiments such as Chromatin Immunoprecipitation sequencing (ChIP-Seq). The application features visualization modules that include time course expression profiles, clustering, gene ontology enrichment analysis, differential expression analysis and comparison of experiments. All visualizations are interactive and interconnected, such that the selection of genes in one module propagates instantly to visualizations in other modules. dictyExpress currently stores the data from over 800 *Dictyostelium* experiments and is embedded within a general-purpose software framework for management of next-generation sequencing data. dictyExpress allows users to explore their data in a broader context by reciprocal linking with dictyBase—a repository of *Dictyostelium* genomic data. In addition, we introduce a companion application called GenBoard, an intuitive graphic user interface for data management and bioinformatics analysis.

**Conclusions:**

dictyExpress and GenBoard enable broad adoption of next generation sequencing based inquiries by the *Dictyostelium* research community. Labs without the means to undertake deep sequencing projects can mine the data available to the public. The entire information flow, from raw sequence data to hypothesis testing, can be accomplished in an efficient workspace. The software framework is generalizable and represents a useful approach for any research community. To encourage more wide usage, the backend is open-source, available for extension and further development by bioinformaticians and data scientists.

## Background

Over seventy five years ago, Dr. Kenneth Raper described the awesome life history of *Dictyostelium discoideum* [1]. This social amoeba grows vegetatively while subsisting on bacteria in the soil, until it exhausts the food supply. Starvation triggers a coordinated process of chemotaxis, aggregation and multicellular development and differentiation of tens of thousands of individual cells. *Dictyostelium*, over the decades, has become a genetic model organism for myriad biological phenomena, including multicellular development, kin recognition, bacterial discrimination and innate immunity [2].

*Dictyostelium* has also been at the leading edge of genomics era research. The genome of *D. discoideum* was among the first eukaryotes to be queued for (Sanger) sequencing [3], and the developmental transcriptome was explored in the early days of gene expression microarrays [4]. Since then, next-generation RNA-sequencing (RNA-seq) has vastly increased the ease and resolution of transcriptome studies [5–7]. And now, researchers are using ChIP-seq to define gene regulatory networks and short-read whole genome sequencing of chemical mutants to dissect genetic pathways [8, 9].

These technological and experimental advances continue to drive the need for new and better approaches to data management and analysis. The sheer volume of NGS output requires data management that is stable and scalable. Scientific best practices dictate that analyses should be rigorous, reproducible and traceable. Software solutions to these challenges typically are designed for data scientists and computational experts. However, these designs often fail to consider the needs, but also the limitations, of many non-computational life scientists who generate and consume the data. To foster the most creative research and efficient collaborative environment, life scientists should be engaged in the entire process; know where their data resides and how it has been processed; and be empowered to explore their data themselves, to ask questions and test hypotheses as they arise.

In collaboration with the *Dictyostelium* group at Baylor College of Medicine, University of Ljubljana developed the original dictyExpress (1.0), a web application designed for exploration of transcriptomics datasets [10]. dictyExpress (1.0) allowed users to select among experiments and specify genes to analyze; visualize the expression time courses of those genes; identify gene clusters; examine pre-processed differential expression datasets; and perform Gene Ontology (GO)-term enrichment analysis.

The distinguishing feature of dictyExpress (1.0) was its interactivity. Each visual analytics module was linked to the others, such that selecting a gene or genes in one module propagated to the others, triggering new analyses where necessary. For example, when the user selected differentially expressed genes in the Volcano Plot, the temporal profiles of these genes appeared in the Time Course module, and GO enrichment terms updated automatically. Gene selection was supported in all visualization modules of dictyExpress, and in this way enabled a variety of workflows and entry points to exploring the data.

The original dictyExpress was developed in Flash (client side) and relied on an *ad-hoc* Python-based backend for data access. Addition of new data was not supported for the user and required manual changes of the database on the server side. End users were precluded from developing new pipelines, as well as tracing the results of bioinformatics analyses. Further, extending the platform to include other species was complicated by inflexibility on the server side.

**Fig. 1:**
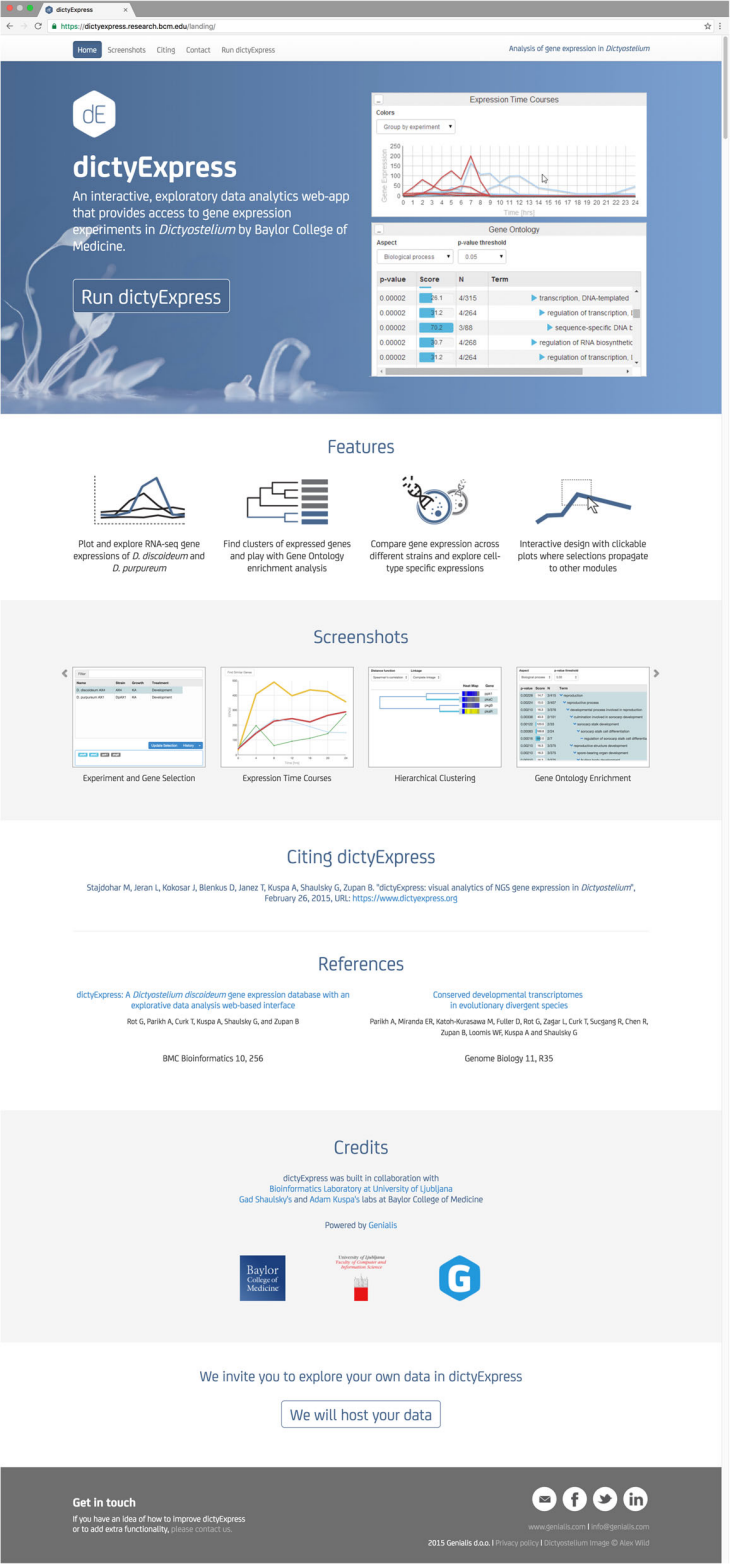
The landing page of the dictyExpress web application invites public and subscribed users. From the URL (dictyExpress.org), this public page provides access to published NGS data

In this paper we report dictyExpress (2.0), a reinvention of the original with an entirely new software architecture and extended functionality (Fig. 1). From the original version [10] we retain the name, several data presentation modalities and the concept of interactive visual exploration. Everything else has changed. The new dictyExpress is bundled with GenBoard, a data management and preprocessing web application. The entire suite has been rewritten in JavaScript, HTML5 and CSS3 on the client side and a high-level Python web framework (Django, version 1.8.6, https://github.com/django/django, https://www.djangoproject.com; PostgreSQL, version 9.4.11, https://github.com/postgres/postgres, https://www.postgresql.org; and MongoDB, version 2.4.8, https://github.com/mongodb/mongo, https://www.mongodb.com) and in-house data flow engine on the server side. The user may now upload raw next-generation sequencing data, trigger the computational pipeline for mapping, estimation of transcript abundance and computation of differential gene expressions, and then use dictyExpress to explore and share the results. Once published, or upon the user’s preferences, results may be marked as public and immediately made available to the general audience.

The new dictyExpress has been adopted as a tool of choice to analyze gene expression data among many prominent labs in the *Dictyostelium* community. As of this submission, the web app has been viewed by over 3700 unique visitors and stores the data from over 800 *Dictyostelium* (and related) experiments. Access to dictyExpress is reciprocally linked to dictyBase, the home page of the central repository for *Dictyostelium* genome data and experimental resources (http://dictybase.org). Every individual gene details page at dictyBase includes a link to dictyExpress, facilitating access to expression profiles, and each gene selection in dictyExpress is linked to the corresponding page in dictyBase. Below, we provide essential details of our implementation framework and describe the functionality of the new dictyExpress. We pay particular attention to the interactive data analysis, and how this feature promotes exploration, discovery and insight generation. We also discuss how the framework could be extended to support other organisms, projects and data types, some of which is already underway.

## Implementation

**Fig. 2:**
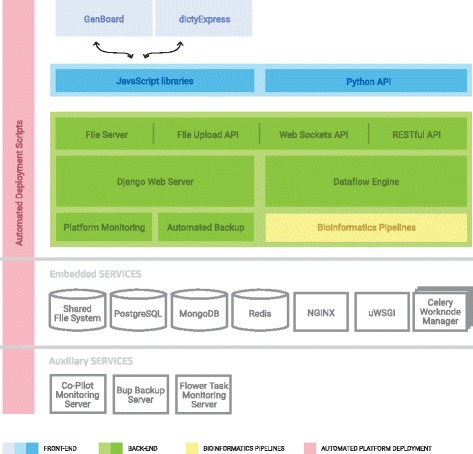
The software behind dictyExpress and GenBoard incorporates a state-of-the art technology stack in a modular framework. The *blue boxes* indicate the user interface layer, with web applications running in JavaScript, and a Python API for programmatic access. The *green boxes* represent the data layer, including the dataflow engine, RESTful API and libraries of bioinformatics tools and pipelines. Beneath these sections are unshaded services layers, including file sharing, database and server systems, and workload managers. The *vertical pink* column represents the glue that connects the various layers and facilitates the seamless interaction between technologies

The dictyExpress web application is part of a larger data analysis software framework (Fig. 2). The backend section of the framework manages the data and executes the analysis pipelines. Data are stored on a file server (raw reads, genomes, ontologies, expressions), MongoDB database (data annotations, links to server files, parameters of analysis pipelines) and PostgreSQL database (data on users and groups, access privileges). Access to the data and analysis pipelines is managed through RESTful API of the Django application framework. This accepts requests from the clients, and schedules analytic tasks to workers. On the client (web browser) side, the framework includes two applications: GenBoard for data and pipeline management, and dictyExpress for interactive analyses. Both GenBoard and dictyExpress are implemented in JavaScript, HTML5 and CSS3, and make use of the following JavaScript libraries: AngularJS, version 1.2.28, (https://angularjs.org/); Bootstrap, version 3.2.0, (http://getbootstrap.com/); c3, version 0.4.10, (http://c3js.org/); d3, version 3.5.5, (https://d3js.org/); and Flot, version 0.8.3, (http://www.flotcharts.org/).

We developed an asynchronous data management platform to trigger different analysis tasks that may depend on results of prior processing steps. The dataflow engine supports defining analysis tasks and dependencies, parallel execution, and status reporting that is used for monitoring on the client side. The GenBoard application is meant to serve data owners and curators as a user interface for the dataflow engine. GenBoard has a familiar dashboard-like layout for data upload, annotation, analysis process automation and monitoring. Meanwhile, the dictyExpress application is responsible for the presentation of results, and serves as the entry point for visualization and exploration. dictyExpress visualizations rely on a chassis of three external libraries—c3, d3, and Flot—which have been extended substantially with interactive capabilities. Our aim was to make all visualization modules interactive and interconnected, such that a user can click a line on a line graph, a branch in a dendrogram, or a dot on a scatter plot, and in this way select the underlying data point. The selection is instantly propagated to all the other modules.

Overall, the implementation codebase includes about 20,000 lines of JavaScript and 30,000 lines of Python. The dataflow and bioinformatics components of the project are open source and available at GitHub (https://github.com/genialis/resolwe).

## Results and discussion

### A new software framework

The redesign and ground-up recoding of the dictyExpress web-application improved the software in numerous ways. From the end-user’s perspective, the interactive data visualizations offer more features and interactivity than before. Thus users can explore many facets of NGS-based gene expression (and ChIP-seq) data more easily. The companion Genboard application facilitates data management and processing, providing tools to ensure traceability and reproducibility of bioinformatics results. Both applications sit atop a framework that enhances data processing performance, and is extensible to virtually any data analysis use-case (Fig. 2).

**Fig. 3:**
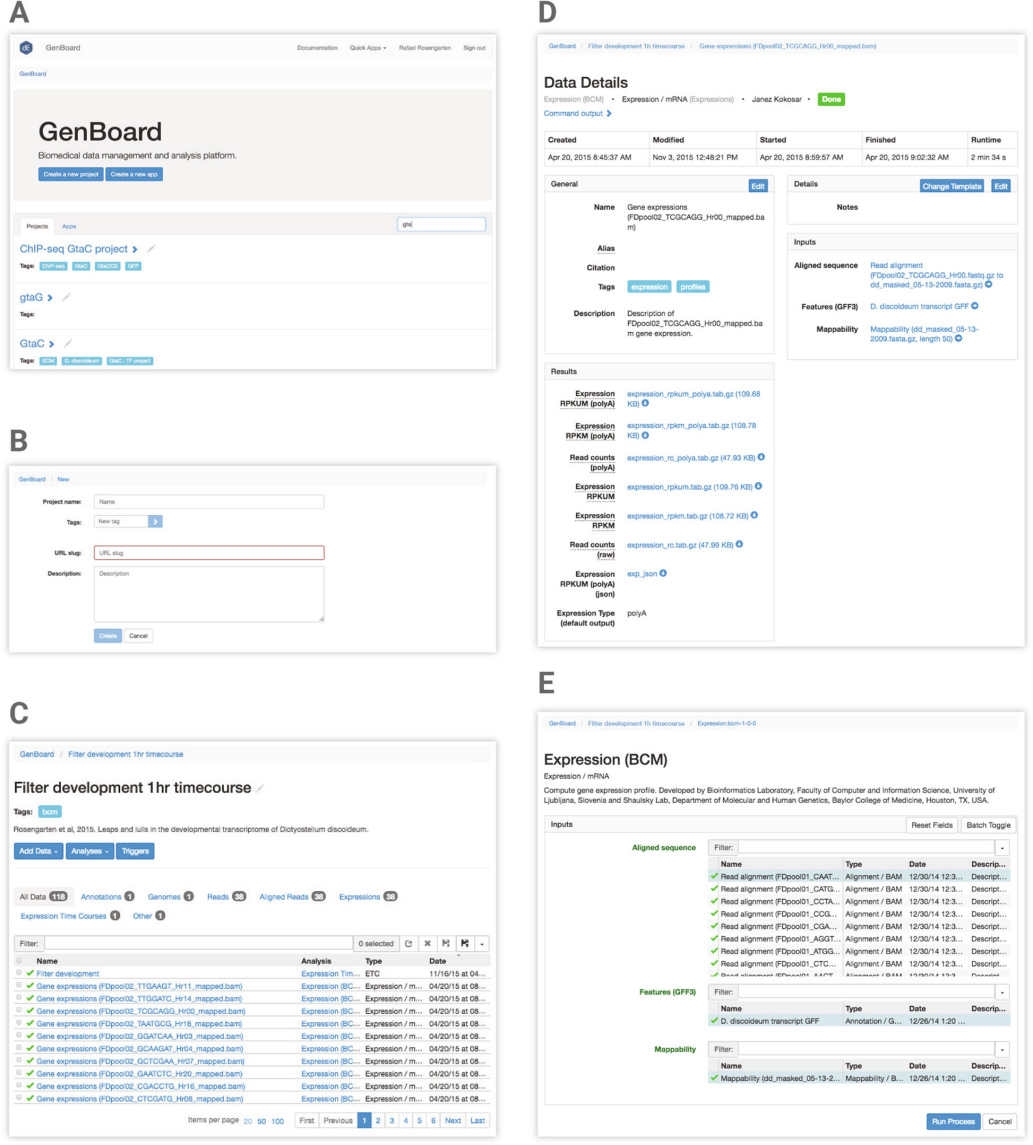
Genboard is the data management graphic user interface. Here users can create a new project, upload raw and processed data files, specify analysis algorithms and parameters, and link one step of the analysis process to another. **a** The user may search/filter among all existing projects based on the project name or descriptive tags. From this page a user may also create a new project (**b**). **c** Within a chosen project, the users find all of the data, input and output files associated with their bioinformatics analysis. These may be filtered by name, type, etc. Clicking on a file name in the table navigates to a data details page (**d**), while clicking on an analysis link in the table navigates to that analysis process (**e**)

Let us illustrate the communication between components of the framework through an example. Consider that a user uploads raw RNA-seq data (e.g. fastq files) with the end goal of displaying gene expression time-course profiles. The user would sign into GenBoard (Fig. 3), upload the raw data and enter the relevant parameters and metadata. The data are transferred to the server and trigger the execution of quality control. Next, through the GUI, the user instructs GenBoard to run mapping and compute gene expression values. These computations run on the server, and, if available, can be distributed over parallel processors to speed-up the execution time. While the computation takes place, GenBoard offers an interface to monitor the progress. Finally, the user can bundle individual data objects, e.g. time-course reads files from sequential biological samples. Upon completion of the computation, the expression data become available on dictyExpress. Access is restricted by default to the author of the data, who may then grant permissions to project partners or make the data public. Any analysis may be shared via the URL.

### Interactive and interconnected visualizations

**Fig. 4:**
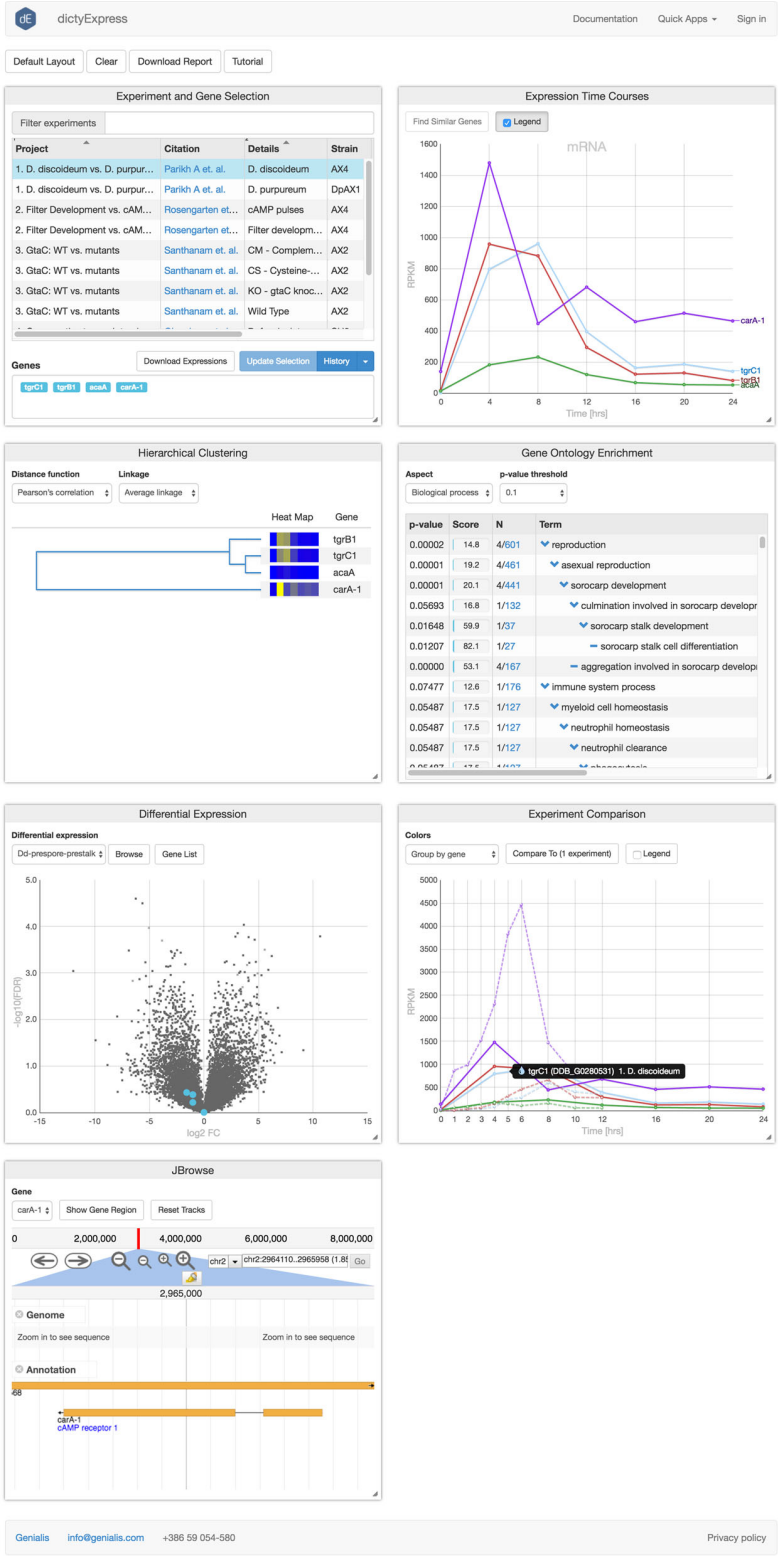
Visual analytics modules of the dictyExpress web application. All modules are interactive and interconnected, such that selections and perturbations in one module propagate to the others
- **Expression Time Courses.** In *Dictyostelium* biology, researchers often explore the changes in gene expression levels over developmental time. In this module, a line graph displays profiles as normalized read count (*y*-axis) versus time (*x*-axis). The *x*-axis scales automatically to accommodate the experimental sampling regime. For studies of non-coding (nc)RNA, selection of these molecules initiates a second line plot with an appropriately scaled *y*-axis [7]. The user can select one or more genes by clicking or dragging across the expression profile curves. Such selections then propagate to all other modules, highlighting data on the selected genes. The user may also discover which genes are most similar to a selected gene. The "Find Similar" pop-up menu enables the user to choose among various methods for scoring of distance between gene profiles. Distances are calculated across the transcriptome in real time, resulting in a table of similar genes that may be appended to the visualization modules. Tool tips provide gene-wise information when the user hovers the mouse over any profile.
- **Hierarchical Clustering.** Genes are clustered based on their expression profiles and the results are shown in a dendrogram, with branches that terminate as heatmaps to illustrate the level of gene expression at different time points. Users may choose one of three methods for distance scoring: Euclidean distance, Pearson’s correlation or Spearman’s correlation, as well as branch linkage criteria. This module allows users to interpret the relative similarity of genes within a gene set, and to select genes for further examination by highlighting selected branches.
- **Gene Ontology Enrichment.** Genes included in the Experiment and Gene Selection module are analyzed for GO term enrichment. The results table includes enrichment statistics and GO terminology. Users may select any of the enriched terms to discover the complete set of associated genes.
- **Differential Expression.** A Volcano Plot is a type of a scatter plot that helps in identification of diffferentially expressed genes. Fold change (FC) is presented on the x-axis (log2 scale), while statistical confidence, derived from the false discovery rate (FDR) increases along the y-axis (^−^ log10FDR). Thus the further any gene sits from zero, the larger the fold change and greater the statistical confidence. The datasets displayed in this module are selected and computed in GenBoard, usually using baySeq [12]. By default, the data available represents differential expression between prespore and prestalk cells, and users may toggle between *D. discoideum* and its sister *D. purpureum* [5]. Genes from the Experiment and Gene Selection module are highlighted in the volcano plot. The user may click or draw a box around any other data points to append to or replace the gene selection. The volcano plot also supports selection of genes from the plot.
- **Experiment Comparison.** The time courses of one or more genes may be compared across different experiments. Users may choose additional experiments to be plotted along with the row-wise selection from the Experiment and Gene Selection module. Time course profiles may be colored by gene or experiment. The same interactivity experienced in the Expression Time Course module applies here.
- **JBrowse.** An implementation of the popular JavaScript genome browser enables viewing gene structure and sequence. JBrowse supports numerous custom tracks, such as ChIP-seq counts [8], non-coding RNA-seq read coverage [7], and WGS variant analysis [9], depending on the experiment and user permissions.

dictyExpress consists of various visual analytics modules. Each module supports the selection of genes—represented by points, lines, branches, etc.—depending on the type of plot (Fig. 4). Gene selections propagate to other modules, are revealed by highlights, and in some cases, trigger new analyses on the fly. Such functionality is referred to as brushing-and-linking [11] and is an essential component of tools for interactive visual analysis. The current dictyExpress includes the following modules:

- **Experiment and Gene Selection.** A table lists in each row projects with available data. Each project is comprised of a collection of read counts pertaining to a particular experiment. For example, a project might include multiple RNA-seq replicates of the wild type strain AX4. The user engages with this module by selecting a project (mouse click), then specifying one or more gene(s) by free text or upload of a gene list text file. Gene inputs, which benefit from auto-complete suggestions, then appear in all other modules. This module also records the work history, allows linking to specific genes in dictyBase and facilitates data downloading.

The JBrowse module and ncRNA sub-module are novel additions relative to the original version of dictyExpress. Besides the new software architecture and entirely rewritten code base, the level of interactivity has also been augmented by including more clickable features and user-controls via pop-up modules.

### Available datasets

dictyExpress showcases published transcriptomics datasets including developmental time courses of *D. discoideum* (AX4) and *D. purpureum* [5]; AX4 development on nitrocellulose filters or during cyclic-AMP pulsing in suspension [6]; and wild type AX2 compared to various AX2 *gtaC* mutant strains [8]. Transcriptomics datasets also extend to taxonomic comparisons between *P. pallidum*, *D. fasciculatum*, and *D. lacteum* [13, 14]. Further, the application hosts the first comprehensive catalog of ncRNA abundance during development [7] and whole genome variant analysis of chemically mutagenized strains [9]. These data will remain open to the community for browsing and exploration. In the future, datasets will become available as they are published.

### Biological insights: real-life example workflow

The principal goal of dictyExpress is to provide biologists, who may not have advanced computational skills, the ability to derive novel insights from high-throughput data. We achieve this by providing the user a set of familiar, interconnected data visualization modules. A biologist may start with a question about the expression pattern of a favorite gene (or genes) in a certain dataset, and proceed by visualizing the gene in other datasets, or by selecting other genes in any of the other modules. Explorations of this type may result in new hypotheses, many of which can be tested in silico prior to wet-lab verification. The visualizations can be captured, saved and communicated to colleagues by copying the URL of any given screen.

**Fig. 5:**
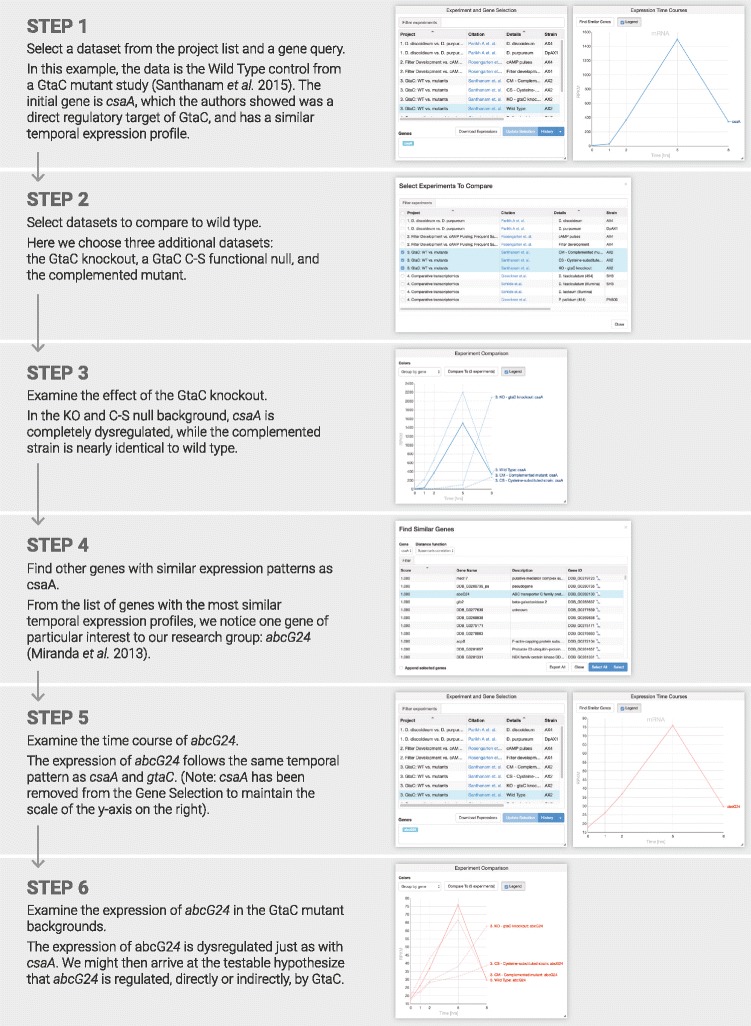
Example dictyExpress workflow. The workflow leads a user from a question to a novel insight and testable hypothesis

In the accompanying example (Fig. 5), we illustrate a simple route to discovering additional candidate target genes of the developmental regulator GtaC [8]. The analysis begins by confirming the GtaC-dependence of the target gene *csaA*, then identifies other genes with similar temporal expression profiles, and finally examines the behavior of one interesting candidate, *abcG24*, in various gtaC^−^ mutant backgrounds. The example illustrates how a researcher may progress from initial knowledge about a gene of interest to a novel, testable hypothesis. Several other examples can be viewed as video animations in the supplemental material, or online at https://www.youtube.com/watch?v=9ayBgHdJMqY.

## Conclusions

New experimental approaches continue to fuel *Dictyostelium* research, and many of these rely on high-throughput sequencing analysis [9, 15]. dictyExpress and GenBoard enable the broad adoption of next generation sequencing based inquiries. The reinvention of dictyExpress yielded an application that is easy to use, addresses many common analysis tasks, and may be extended to meet future needs. The inclusion of GenBoard offers biologists a solution for or data management and processing, to complement the exploratory analyses of dictyExpress. The entire information flow, from raw sequence data to hypothesis testing and novel insights, can now be accomplished in an intuitive and efficient workspace.

The new system architecture and technology stack are designed to evolve to keep pace with experimental, sequencing, and bioinformatics advances. We envision an ongoing process of improvement as technology and users demand. Already we are eyeing updates such as providing programmatic access via API for data management and bioinformatics support that will appeal to data experts. We also plan to expand bioinformatics support and dataflow capabilities by leveraging open source contributions.

## Abbreviations

ChIP-seq: Chromatin immunoprecipitation sequencing
FC: Fold change
FDR: False discovery rate
GO: Gene ontology
ncRNA: non-coding RNA
RNA-seq: RNA sequencing
WGS: Whole genome sequencing

## References

[1] Raper K (1940). Pseudoplasmodium formation and organization in *dictyostelium discoideum*. J Elisha Mitchell Sci Soc.

[2] Williams J (2010). *Dictyostelium* finds new roles to model. Genetics.

[3] Eichinger L, Pachebat JA, Glockner G, Rajandream MA, Sucgang R, Berriman M, Song J, Olsen R, Szafranski K, Xu Q, Tunggal B, Kummerfeld S, Madera M, Konfortov BA, Rivero F, Bankier AT, Lehmann R, Hamlin N, Davies R, Gaudet P, Fey P, Pilcher K, Chen G, Saunders D, Sodergren E, Davis P, Kerhornou A, Nie X, Hall N, Anjard C, Hemphill L, Bason N, Farbrother P, Desany B, Just E, Morio T, Rost R, Churcher C, Cooper J, Haydock S, van Driessche N, Cronin A, Goodhead I, Muzny D, Mourier T, Pain A, Lu M, Harper D, Lindsay R, Hauser H, James K, Quiles M, Madan Babu M, Saito T, Buchrieser C, Wardroper A, Felder M, Thangavelu M, Johnson D, Knights A, Loulseged H, Mungall K, Oliver K, Price C, Quail MA, Urushihara H, Hernandez J, Rabbinowitsch E, Steffen D, Sanders M, Ma J, Kohara Y, Sharp S, Simmonds M, Spiegler S, Tivey A, Sugano S, White B, Walker D, Woodward J, Winckler T, Tanaka Y, Shaulsky G, Schleicher M, Weinstock G, Rosenthal A, Cox EC, Chisholm RL, Gibbs R, Loomis WF, Platzer M, Kay RR, Williams J, Dear PH, Noegel AA, Barrell B, Kuspa A (2005). The genome of the social amoeba Dictyostelium discoideum. Nature.

[4] Van Driessche N, Shaw C, Katoh M, Morio T, Sucgang R, Ibarra M, Kuwayama H, Saito T, Urushihara H, Maeda M, Takeuchi I, Ochiai H, Eaton W, Tollett J, Halter J, Kuspa A, Tanaka Y, Shaulsky G (2002). A transcriptional profile of multicellular development in Dictyostelium discoideum. Development.

[5] Parikh A, Miranda ER, Katoh-Kurasawa M, Fuller D, Rot G, Zagar L, Curk T, Sucgang R, Chen R, Zupan B, Loomis WF, Kuspa A, Shaulsky G (2010). Conserved developmental transcriptomes in evolutionarily divergent species. Genome Biol.

[6] Rosengarten RD, Santhanam B, Fuller D, Katoh-Kurasawa M, Loomis WF, Zupan B, Shaulsky G (2015). Leaps and lulls in the developmental transcriptome of Dictyostelium discoideum. BMC Genomics.

[7] Rosengarten RD, Santhanam B, Kokosar J, Shaulsky G (2017). The long non-coding RNA transcriptome of *dictyostelium discoideum* development. G3: Genes | Genomes | Genetics.

[8] Santhanam B, Cai H, Devreotes PN, Shaulsky G, Katoh-Kurasawa M (2015). The GATA transcription factor GtaC regulates early developmental gene expression dynamics in *Dictyostelium*. Nat Commun.

[9] Li CL, Santhanam B, Webb AN, Zupan B, Shaulsky G (2016). Gene discovery by chemical mutagenesis and whole-genome sequencing in *Dictyostelium*. Genome Res.

[10] Rot G, Parikh A, Curk T, Kuspa A, Shaulsky G, Zupan B (2009). dictyExpress: a *Dictyostelium* discoideum gene expression database with an explorative data analysis web-based interface. BMC Bioinforma.

[11] Ward M, Grinstein G, Keim D (2010). Interactive Data Visualisation.

[12] Hardcastle TJ, Kelly KA (2010). baySeq: empirical Bayesian methods for identifying differential expression in sequence count data. BMC Bioinforma.

[13] Schilde C, Lawal HM, Noegel AA, Eichinger L, Schaap P, Glockner G (2016). A set of genes conserved in sequence and expression traces back the establishment of multicellularity in social amoebae. BMC Genomics.

[14] Glockner G, Lawal HM, Felder M, Singh R, Singer G, Weijer CJ, Schaap P (2016). The multicellularity genes of dictyostelid social amoebas. Nat Commun.

[15] Zhang X, Zhuchenko O, Kuspa A, Soldati T (2016). Social amoebae trap and kill bacteria by casting DNA nets. Nat Commun.

